# Hemichorea Associated With Non-ketotic Hyperglycemia: A Case Report and Literature Review

**DOI:** 10.3389/fneur.2020.00096

**Published:** 2020-02-25

**Authors:** Wei Zheng, Lin Chen, Jian-hao Chen, Xiang Lin, Yi Tang, Xiao-juan Lin, Jing Wu, Zhao-min Lin, Jing-yuan Lin

**Affiliations:** ^1^Department of Neurology, Fujian Provincial Geriatric Hospital, Fuzhou, China; ^2^Department of Internal Medicine, Fujian Provincial Hospital South Branch, Fuzhou, China; ^3^Department of Rehabilitation, Fujian Provincial Hospital, Fuzhou, China

**Keywords:** non-ketotic hyperglycemia, hemichorea, magnetic resonance imaging, lentiform nucleus, blood glucose

## Abstract

**Objective:** To explore the clinical manifestation, diagnosis, therapy, and mechanism of hemichorea associated with non-ketotic hyperglycemia (HC-NH) so as to enhance awareness and avoid misdiagnosis or missed diagnosis of the disease.

**Methods:** A case of HC-NH was reported and reviewed in terms of the clinical features, diagnosis and treatment.

**Results:** Hemichorea associated with non-ketotic hyperglycemia is a rare complication of diabetes mellitus, which is commonly seen in elderly women with poorly-controlled diabetes. The condition is characterized by non-ketotic hyperglycemia, unilateral involuntary choreiform movements, and contralateral basal ganglia hyper-intensity by T1-weighted MR imaging or high density on CT scans. Blood glucose control is the basal treatment, in combination with dopamine receptor antagonists and benzodiazepine sedative, in controlling hemichorea.

**Conclusion:** In clinical practice, the possibility of unilateral chorea should be considered for diabetic patients with poor blood glucose control.

## Introduction

Hemichorea is usually associated with a contralateral lesion in the central nervous system and can result from infection, genetic mutation, neoplasms, neurodegeneration, stroke, metabolic disease, drug-exposure, and autoimmune disease (1, 2). Hemichorea associated with non-ketotic hyperglycemia (HC-NH) is a rare complication of diabetes mellitus (3), which is commonly seen in elderly diabetic women with poor blood glucose control. HC-NH was first described in 1960 (4) and is characterized by non-ketotic hyperglycemia, unilateral involuntary choreiform movements, and contralateral basal ganglia hyper-intensity on T1-weighted MR images or high density on CT scans (5).

According to a meta-analysis, the average age of HC-NH patients is 71 years old, with a male-to-female ratio of 1–1.8 (6). HC-NH mostly occurs in non-ketotic diabetic patients with poor blood glucose control in the past (3), sometimes in ketotic diabetic patients (7), and occasionally in adolescents with newly-diagnosed diabetes (8). Most of the hemichorea is found in the course of diabetes in HC-NH patients, and some of the first symptoms precede the discovery of diabetes (9). In general, dance movements usually occur on the upper and lower limbs of the ipsilateral side and rarely on both sides of the body, which are characterized by rapid, involuntary, and irregular dancing of the limbs, partially involving the muscles of the face and neck, and accompanied by eyebrow extrusion, mouth skimming, tongue extension and other symptoms. Chorea becomes obvious when the person is in an emotional mood and can disappear after sleep (10). With the control of blood glucose, the imaging lesions can be gradually absorbed and dissipated (11). But the pathogenetic mechanism of HC-NH during hyperglycemia and the nature of the neuroradiological findings remain unclear.

The current paper reports an HC-NH case and reviews the clinical characteristics, pathophysiological mechanism, imaging features, treatment, and prognosis of HC-NH.

## Case Report

A 58-year-old woman, admitted on January 11, 2019, complained of a 2-month history of continuous involuntary choreic movements of her right leg. The right lower limb was in an abduction position and the toes were in constant flexion and stretching to a great extent, which resembled an aimless, irregular, rapid, and non-autonomous movement. The movement was non-suppressible and ceased only during sleep. As the condition did not affect her trunk, other limbs, or her face and no other conditions were evident such as visual complaints, giddiness, limb weakness, facial deviation, slurred speech, squinting, or double vision, the patient ignored the symptoms and received no treatment. On January 3, 2019, the autonomic movement of the right lower limb aggravated with increased frequency and amplitude, though the nature and form of movements remained the same. The patient visited the Department of Neurology of the Affiliated Union Hospital of Fujian Medical University and was diagnosed with “hemichorea.” The above symptoms were not relieved after the patient took the prescribed “oxcarbazepine and trihexyphenidyl” irregularly for 3 days (Supplementary Videos 1, 2).

**Figure 1:**
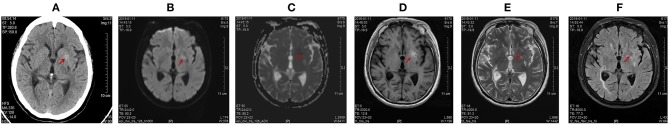
Computed tomography (CT) of the brain on January 15, 2019 and magnetic resonance imaging (MRI) of the brain on January 11, 2019. Abnormal signals were marked by red arrow. **(A)** The unenhanced high-density axial CT images within the left lentiform nucleus. The range of this abnormal signal was about 11.4 mm * 10.6 mm, and the CT value was 50.7 Hu. **(B)** The left lentiform nucleus showed a slightly high signal intensity by diffusion weighted imaging (DWI). **(C)** The left lentiform nucleus showed an equal signal intensity on ADC images. The ADC value of the abnormal lesion center was 0.636 * 10^−3^ mm^2^/s. **(D)** The left lentiform nucleus with a high signal intensity on T1-weighted MR images. The range of this abnormal signal is about 21 mm * 11 mm. **(E)** The left lentiform nucleus showed a low signal intensity on T2-weighted scans. **(F)** The left lentiform nucleus indicated a low signal intensity on fluid-attenuation inversion recovery (FLAIR) sequences.

The patient has been afflicted with type 2 diabetes for more than 20 years. She has taken metformin and acarbose combined with subcutaneous injection of Novomix30 and glargine insulin, but the blood glucose was not appropriately controlled. In addition, she had a history of hypertension and has received hysterectomy and minimally invasive surgery for left kidney stones. Motor examination revealed nearly continuous low-amplitude choreoathetosis in the right lower limb but no evidence of bradykinesia or rigidity. No other remarkable neurological abnormalities were noted and no muscle weakness was present in either the upper or the lower limbs. The examinations of dysarthria, postural instability ataxia and myoclonus reported negative results. Laboratory examinations reported normality in full blood count, liver function tests, renal function tests, serum ceruloplasmin, tumor markers, inflammatory markers, thyroid function tests, urine protein/creatinine ratio, electrocardiogram, and electroencephalogram. In addition, the reading of her thyroid functions fell within the normal range and that of serum thyroid autoantibodies was negative. Similar negative results were observed for collagen disease antinuclear antibodies, antiphospholipid antibody, AIDS antibody, and TPPA. Her blood glucose was 12.17 mmol/L (normal range: 3.9–6.1 mmol/L) with no ketone bodies in the urine on admission but her hemoglobin A1C reading was 14.5% (normal range 4.27%−6.07%). Radiological images on admission showed left lentiform nucleus abnormality with typically high hyperattenuation on computed tomography (CT) scan and hyperintensity on T1-weighted magnetic resonance imaging (MRI) (Figure 1D). No gene variation was found that may be related to clinical phenotype/initial diagnosis.

**Figure 2:**
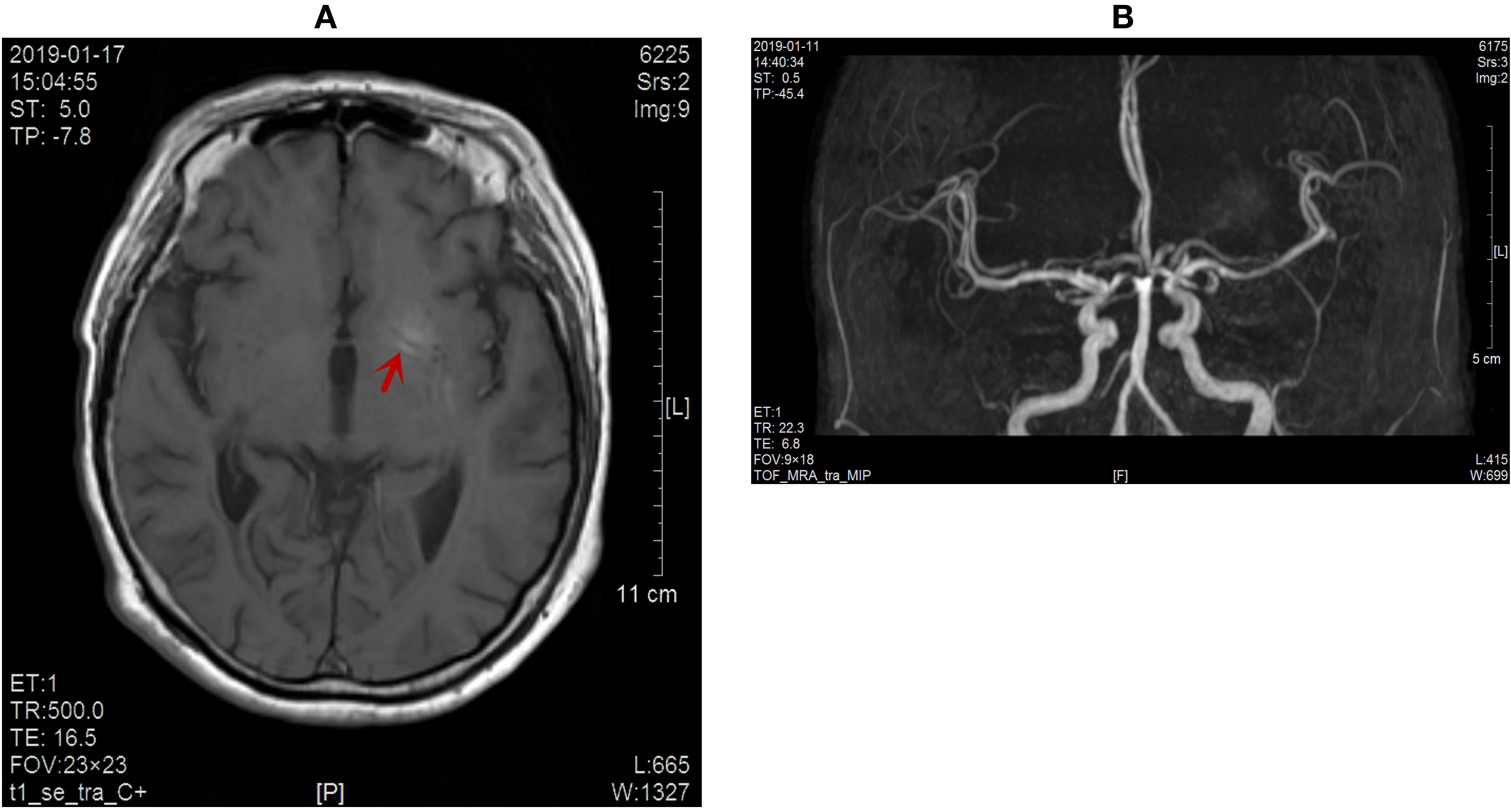
**(A)** No obvious enhancement of the lesion on contrast-enhanced MR images (red arrow). **(B)** No abnormal blood vessel shadow observed on the skull MRA scans.

The patient developed an involuntary dance-like movement in the right lower limb, which was a symptom of the extrapyramidal system located in the left basal ganglia. In combination with the results of skull MR, the responsible focus was the lenticular nucleus. The serum magnesium, calcium, and thyroid function of the patient were normal, and the chorea caused by metabolic disorders such as hyperthyroidism, hypocalcemia, and hypomagnesemia was excluded. The patient had no individual or familial history of liver disease. The serum ceruloplasmin was normal and no KF ring was found in the cornea, so hepatolenticular degeneration was excluded. The patient reported a chronic onset, no history of infection, and no other discomfort such as fever, headache, vomiting, mental disorder, and negative meningeal irritation. HIV antibody, syphilis antibody, erythrocyte sedimentation rate (ESR) and anti-O antibody were normal, and acquired chorea caused by virus, bacteria (streptococcus), syphilis, AIDS and other pathogens were excluded. The patient had no rapidly progressive dementia and no motor disorders such as myoclonus. EEG was normal upon admission, and no hockey sign or lace sign was found in skull MR, so Ruan albuminosis was excluded. Because no abnormal blood vessel shadow was evident in the examination of skull MRA (Figure 2B), the imaging changes of skull MR did not fit the time evolution of cerebral hemorrhage and cerebral infarction, which ruled out the possibility of acquired chorea caused by vascular diseases. The patient had normal immune indices and no discomfort such as dry mouth, dry eyes, rash, joint swelling, and pain, so chances of immune diseases such as systemic lupus erythematosus and antiphospholipid syndrome were ruled out. Drug-induced chorea was excluded as the patient was not exposed to drugs. No abnormal enhancement of the lesions was found in the enhancement of skull MR, and the intracranial tumors were excluded (Figure 2A). No genetic variation associated with clinical phenotype/initial diagnosis was found in monogenic disease screening. Huntingdon's disease was excluded due to no family history of the disease. The patient was an elderly female diabetic patient with poor blood glucose control, and the urinary ketone body and blood ketone body were negative at the time of onset. Her clinical manifestation was unilateral gradual aggravation of dance-like autonomic movement. The left striatum of the responsible lesion showed a high-density shadow in skull CT scans and high signal intensity in T1-weighted MR images. Therefore, her condition was diagnosed as hemichorea associated with non-ketotic hyperglycaemia.

During hospitalization, the blood glucose was actively controlled, and the extrapyramidal symptoms were improved with the administration of haloperidol and trihexyphenidyl. Meanwhile, after the treatment with nerve-nurturing drugs and antioxidation, the symptom of the involuntary dance-like movement in the right lower limb was gradually improved. The patient underwent a reexamination of skull CT and skull MR in our hospital on January 29 and April 17, 2019, respectively. After discharge, the patient actively controlled her blood glucose (pre-meal blood glucose was controlled in 7–8 mmol/L and the post-prandial blood glucose was controlled at 8.5–10 mmol/L), and gradually reduced the dosage of haloperidol. During the follow-up on June 1, the patient reported no more autonomous dance-like movement in the right lower limb in the week after discharge. Hemichorea did not relapse during a 1-month follow-up.

### Image Data

SOMATOM Definition 64 multi-detector CT was used with the parameters of the helical scan method set as follows: 120 kVp, 1 s of rotation time, 5 mm of slice thickness and increment, 250 mm of FOV, 512 × 512 of matrix size, 64 × 0.625 mm of collimation, and 1 of pitch. An united-imaging uMR560 (1.5T) scanner with an 8-channel body phase-array coil was employed. The imaging parameters were set as follows: TE/TR: 13.04/2,000 ms; FOV: 20 cm × 23 cm; Matrix: 128 × 128; Slicethic: 5 mm; and Nex: 8. The diffusion–weighted images (DWIs) were obtained in the transverse plane by means of a single-shot echo planar image (TR/TE: 6,500/125 ms; FOV: 24 × 24 cm; Matrix: 128 × 128; Slicethick: 5 mm; and 2 b values, 0 and 1,000 s/mm^2^). Apparent diffusion coefficient (ADC) maps were calculated on apixel-by-pixel basis.

## Discussion

### Pathophysiological Mechanism of HC-NH

Currently, several hypotheses have been proposed at home and abroad to illuminate the pathogenesis of HC-NH:

1. **Metabolic disorder theory:** When HC-NH patients are in a state of hyperglycemia, the brain tissue obtains energy by anaerobic metabolism and the tricarboxylic acid cycle is suppressed. At this moment, the energy source for brain cells is gamma-aminobutyric acid (GABA), which can be synthesized by acetoacetic acid in ketosis patients, but not in non-ketosis patients after GABA is rapidly depleted. This can disturb the neurotransmitter balance, facilitate thalamic-cortical feedback, and eventually lead to dance-like extrapyramidal symptoms (8). According to this theory, chorea should be bilateral, but most chorea cases in HC-NH patients are unilateral, so the theory cannot fully explain the pathogenesis of HC-NH.
2. **Ischemic injury or ion deposition theory:** This school of thought argues that the high signal of T1-weighted MR images in these patients is caused by ischemic injury (12), which leads to the proliferation of astrocytes and the expression of zinc-friendly metalloproteins (13). It will also lead to the deposition of paramagnetism, which will eventually lead to the increased signal in the lesion area. Some scholars (14) have also found that local ischemic injury can induce the accumulation of manganese ions in rat striatum astrocytes, resulting in paramagnetism.
3. **Hemorrhagic injury theory:** Nath et al. (15) reported trace bleeding in the focus of HC-NH patients. However, the hemorrhage is different from the general cerebral hemorrhage, in that the hematoma is limited to the lenticular nucleus and putamen nucleus, and does not oppress the internal capsule and adjacent structures, without edema, and space occupying effect. Moreover, some scholars failed to find any evidence of hemosiderin deposition in the autopsy of the disease (16). Therefore, the theory of microhemorrhage in striatum or hemorrhage after infarction cannot fully explain the mechanism of the disease.
4. **Dopaminergic and estrogenic theory:** When the blood glucose metabolism is disturbed, the level of dopaminergic (DA) increases, which can enhance the facilitation of the direct loop in the basal nucleus neural loop and weaken the inhibitory effect of the indirect loop. This will make it impossible for the basal nucleus nerve loop to regulate normal movement and induce hemichorea (17). The clinical symptoms of this patient were significantly improved with the control of blood glucose and the use of dopamine receptor antagonist haloperidol. Our hypothesis is that hyperglycemia may directly lead to changes in dopaminergic activity in the striatum of predisposed patients with dopamine receptors up-regulated and DA catabolic metabolism decreased (11). Moreover, the depletion of GABA, used as an alternate energy substrate during hyperglycemic crises, may cause a decreased inhibition of the thalamus by the medial part of the globus pallidus (18). The combination of decreased thalamic inhibition and a recent or old striatal lesion, which may increase the inhibition of the subthalamic nucleus, may be responsible for contralateral HC-NH (19). Estrogen can antagonize the function of DA in the substantia nigra striatum system, which leads to the activation of DA receptor in the striatum system, which is easy to induce autonomic movement (7). Therefore, as reported in the current study, most of these diseases occur in elderly women.
5. **Autoimmune inflammatory response theory:** Ahlskog et al. (20) found that the titer of anti-glutamic acid decarboxylase (GAD) in the striatum of some HC-NH patients was significantly increased. It is suggested that GAD-mediated autoimmune injury is also involved in HC-NH.
6. **Neurodegeneration theory:** The disorder of glycometabolism impairs the demyelination of nerve fibers. It can cause Waller degeneration of the white matter in the striatum under the action of a high osmotic pressure or hyperglycemia (21). Axonal dehydration during this process can explain the temporal dynamic imaging changes of CT and MR T1WI images.

Altogether, we speculate that the pathogenesis of HC-NH involves the altered GABA and dopaminergic neurotransmission.

### Imaging Features of HC-NH

**Figure 3:**
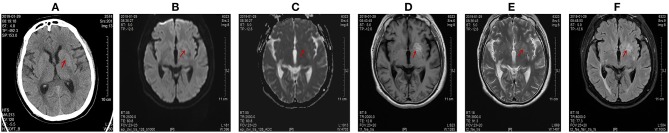
Computed tomography (CT) scans and magnetic resonance imaging (MRI) images of the brain on January 29, 2019. Abnormal signals were marked by red arrow. **(A)** The disappearance of the high signal intensity in the left lentiform nucleus on CT scans. The CT value of abnormal lesion center was 36.9 Hu. **(B)** The left lentiform nucleus showed an equal signal intensity on DWI scans. **(C)** The left lentiform nucleus showed an equal signal intensity on ADC images. The ADC value of the abnormal lesion center was 0.805 * 10^−3^ mm^2^/s. **(D)** The left lentiform nucleus showed a high signal intensity on axial T1-weighted images, but the range was slightly extended. The range of this abnormal signal was about 22.0 mm * 12.4 mm**. (E)** The lesion of the left lentiform nucleus changed from low signal to high signal on T2-weighted scans. **(F)** The lesion of the left lentiform nucleus changed from low signal to high signal on T2-FLAIR images.

**Figure 4:**
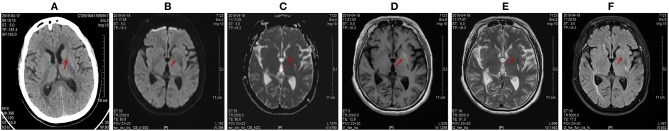
Computed tomography (CT) scans the brain on April 17, 2019 and magnetic resonance imaging (MRI) images of the brain on April 18, 2019. Abnormal signals were marked by red arrow. **(A)** The disappearance of the high signal intensity in the left lentiform nucleus on CT scans. The CT value of the abnormal lesion center was 32 Hu. **(B,C)** The left lentiform nucleus showed an equal signal intensity on DWI and ADC scans. The ADC value of the abnormal lesion center was 0.846 * 10^−3^ mm^2^/s. **(D)** The lighter and narrowed signal intensity of the left lentiform nucleus on T1-weighted images. The abnormal signal range of the left lentiform nucleus was narrowed to 8 mm * 4 mm. **(E,F)** The disappearance of the original high signal intensity on T2-weighted and T2-FLAIR images.

The CT findings of HC-NH showed a high density in the contralateral striatum of the affected limb (Figure 1A), but the high-density lesion disappeared in a short period of time (1–6 months). MR imaging revealed a high signal in T1-weighted MR images, slightly low signal or equal signal for T2WI, and low signal for DWI. FLAIR sequencing showed a dominance of equal or low signal and a minority of mixed high-and-low signal, and enhanced scan reported no enhancement (13, 22). Single photon emission computed tomography (SPECT) showed a significant decrease in blood perfusion at the corresponding lesions (23). After 3 months of treatment, we found a lighter and narrowed signal intensity in the left lentiform nucleus on T1-weighted images on April 18, 2019 (Figure 4D). The abnormal signal range of the left lentiform nucleus was narrowed to 8 mm ∗ 4 mm. The ADC maps indicated restrictions of the diffusion of molecules by structures such as cell membranes, and reflected the microstructure of the cellular environment. DW-MRI is sensitive to changes in the diffusion of water molecules. Therefore, ADC value of the lesion center was chosen as a quantitative index to reflect the changes in the image. The ADC value of the abnormal lesion center was 0.636 ∗ 10^−3^ mm^2^/s (Figure 1C). With the control of blood glucose and the improvement of clinical symptoms, the ADC value of the abnormal lesion center increased to 0.805 ∗ 10 ^−3^ mm ^2^/s (Figure 3C) and 0.846 ∗ 10 ^−3^ mm^2^/s (Figure 4C). This is consistent with the view of some scholars that the apparent diffusion coefficient (ADC) of (DWI) can be used to judge the involved area and prognosis of HC-NH patients (24). This case also confirms that with the control of blood glucose, the imaging lesions can be gradually absorbed and dissipated. In short, the imaging changes came later than the improvement of clinical symptoms.

### Treatment and Prognosis of HC-NH

By actively controlling blood glucose and using dopamine receptor antagonists such as haloperidol, most HC-NH patients have a good prognosis (25), which is also confirmed in the current study. It is important to note that because some patients may develop side effects such as tremors from taking dopamine receptor antagonists, we should start with a small dose and slowly increase the dose and adopt an individualized treatment scheme. Meanwhile, a high treatment compliance should be maintained in the process of treatment to avoid recurrence after symptom remission. Some studies (26, 27) have also found that the use of ventral thalamus, globus pallidus incision, or implantation of deep brain stimulation system in the ventral anterior nucleus of the affected thalamus can also effectively control dance symptoms in patients with refractory HC-NH.

## Conclusion

In summary, although HC-NH is rare in clinical settings, we should be alert to the potential presence of the disease in case of unilateral chorea in diabetic patients with poor blood glucose control. Timely skull imaging examination and active blood glucose control can avoid misdiagnosis and delay in treatment.

## Data Availability Statement

The raw data supporting the conclusions of this article will be made available by the authors, without undue reservation, to any qualified researcher.

## Ethics Statement

The studies involving human participants were reviewed and approved by this study was approved by Ethics Review Board of Fujian Provincial Geriatric Hospital. The patients/participants provided their written informed consent to participate in this study. Written informed consent was obtained from the individual(s) for the publication of any potentially identifiable images or data included in this article.

## Author Contributions

WZ and LC wrote the manuscript. JC, XianL, YT, XiaoL, JW, ZL, and JL critically revised the manuscript. All authors read and approved the submitted version.

### Conflict of Interest

The authors declare that the research was conducted in the absence of any commercial or financial relationships that could be construed as a potential conflict of interest.

## Supplementary Material

Supplementary Videos 1 and 2 Recorded the clinical manifestations of patients in the Supplementary Materials.
